# Biomedical Vibrational Spectroscopy in the Era of Artificial Intelligence

**DOI:** 10.3390/molecules26051439

**Published:** 2021-03-06

**Authors:** Henry Horst Mantsch

**Affiliations:** Independent Researcher, Ottawa, ON K2G-5X9, Canada; henry.mantsch@rogers.com

**Keywords:** medical infrared and Raman spectroscopy, artificial intelligence, biomedical informatics, diagnosis of disease

## Abstract

Biomedical vibrational spectroscopy has come of age. The past twenty years have brought many advancements and new developments and now its practitioners face a new challenge: artificial intelligence. Artificial intelligence has the capability to detect meaningful relationships in data sets such as those found in an infrared or Raman spectrum. The present narrative assesses the degree to which biomedical vibrational spectroscopy has already embraced artificial intelligence and what can be expected going forward. This article belongs to the Special Issue Biomedical Applications of Infrared and Raman Spectroscopy.

## 1. Introduction

Molecular spectroscopists have been practicing biomedical infrared and Raman spectroscopy for some time, yet it remains a somewhat undervalued field of study. As interest in this type of spectroscopy is steadily growing, it is appropriate to devote an entire issue of this journal to this topic. Concurrently we are witnessing significant developments in a different domain of human endeavor, namely that of artificial intelligence. In fact, the rise of artificial intelligence is often compared to the Industrial Revolution of the 18th century, which ushered in sweeping social and technological change. Artificial intelligence is rapidly expanding its reach into all spheres of human life. It has revolutionized science, unleashing powerful new tools for making sense of the large data sets generated by our scientific technology. Biomedical vibrational spectroscopy stands perfectly poised to take advantage of developments in artificial intelligence. The objective of this narrative is to comment on the degree to which artificial intelligence has affected biomedical vibrational spectroscopy so far and where this may lead in the future.

## 2. The Past: Where did We Come From?

The origins of vibrational spectroscopy go back to the detection of infrared radiation in 1801 and the discovery in 1928 of what is now referred to as the Raman effect. Each of these discoveries was followed by important developments, yet vibrational spectroscopy, as we know it today, only came into its own in the middle of the 20th century.

Major advancements, especially in infrared spectroscopy had occurred during WWII, but these discoveries were made under wartime restrictions and only became public knowledge much later. Following the devastating WWII conflict chemists from all over the world discovered (some even re-discovered) infrared and Raman spectroscopy, employing it for the determination of many new molecular structures, as well as for a variety of other chemical applications. 

A milestone in the history of vibrational spectroscopy was the publication in 1945 of Gerhard Herzberg’s seminal treaty *“Infrared and Raman Spectra of Polyatomic Molecules*” [1]. Herzberg’s lifelong work validated the integral use of vibrational spectroscopy in chemistry, earning him the 1971 Nobel Prize for Chemistry.

With the routine use of lasers for Raman spectroscopy after 1963 and the introduction of interferometric infrared spectrometers in the late 1970s, vibrational spectroscopy became more powerful and henceforth could be deployed for the investigation of more complex molecules. The emphasis gradually shifted from chemistry to biology. *Biomolecular vibrational spectroscopy* now was able to tackle the watery world of biology.

Then, throughout the 1990th, bio-spectroscopists began to apply their tools of infrared and Raman spectroscopy to the study of human body fluids, cells and tissues and by the turn of the century biomedical vibrational spectroscopy was established as an independent area of research.

Artificial intelligence, on the other hand, had a more checkered history. Its origins can be traced back to Alan Turing who speculated back in the 1950s about “thinking machines” that could reason at the level of a human being. However, it was John Mc Carthy, a computer science pioneer, who first used the term “artificial intelligence” to denote machines that could think autonomously. This engendered much idealistic hype about artificial intelligence (AI), but as the high expectations did not materialize, a so called “AI winter” set in, with computer scientists and software engineers avoiding the term artificial intelligence for fear of being viewed as dreamers.

Artificial intelligence was still growing slowly throughout the first decade of the 21st century, but once it became accepted outside the community of computer scientists the applications of AI picked up speed and are now spreading like wildfire. Artificial intelligence tops the list of current technological buzzwords; we had the personal computer (PC) in the 1980s, Internet and World Wide Web in the 1990s, smart phones and social media in the 2000s and now artificial intelligence and machine learning. 

## 3. Vibrational Spectroscopy Encounters AI

When we compare the rise of AI with the industrial revolution, we must remember that the industrial revolution was spurred on by new technological developments like the steam engine. These technical advances, in combination with plenty of raw materials such as coal and cheap labor, were driving the industrial revolution. The artificial intelligence revolution on the other hand, is data driven. The new raw materials for AI are big data. Vast quantities of digital numbers are the most precious commodity for AI, which, combined with the rapidly increasing computing power, drive the AI revolution. Infrared and Raman spectra are comprised of hundreds or thousands of individual frequencies per spectrum, providing an ideal collection of data to be exploited by AI. Using the capability of AI algorithms to uncover meaningful relationships in such data sets as are found in an infrared or Raman spectrum we can extract very small correlated spectral differences that are often smaller than the uncorrelated noise in the vibrational spectra. 

Before vibrational spectroscopy could fully take advantage of AI, the former first had to “*go digital”.* Let us not forget that initially vibrational spectra were obtained as paper traces with the wavelengths (frequencies or wavenumbers) on the *x*-axis and the intensities on the *y*-axis. The spectrometers were analog devices operating by means of mechanical or electrical servo-mechanisms. In the late 1950s impressive, but cumbersome digital computers were beginning to appear in academic and industrial institutions. These massive computers were usually housed in their own buildings with their own support staff. Their impact on spectroscopy was slow to catch on, with many analytical spectroscopists reluctant to “go digital”. Then, in the 1960s two pioneers, Norman Jones at the NRC in Ottawa and Abe Savitzky at Perkin-Elmer in Connecticut started the electronic revolution in infrared spectroscopy by “marrying infrared spectroscopy to the computer”. In the 1960s when this author joined the lab of Dr Jones in Ottawa, our spectroscopic data were still transferred to the NRC mainframe computer by punching holes on color-coded IBM cards [2]. It was a slow business, prone to typographical errors, but this approach also had a bright side. The debris collected from the punching was in demand as a substitute for confetti on festive occasions, especially weddings. It was less popular with the ecclesiastical authorities as it clung more strongly than regular confetti and was hard to sweep up.

Subsequently, numerical data recording moved from punched IBM cards to punched paper strips and by the late 1960s the Perkin-Elmer 521 spectrophotometer already had provision for digital data readout on punched paper tape. In short order data recording moved to magnetic tapes, magnetic disks and then on to other more sophisticated recording and storage devices, culminating with the latest “cloud storage”. We have reached the point where the wealth of information contained in the experimental infrared and Raman spectra can be easily harvested and shared globally.

If one had to identify a single most important development in the history of biomedical vibrational spectroscopy it would be the emergence of bioinformatics and medical informatics. Today biomedical vibrational spectroscopy relies heavily on mathematical methods for data mining, so following the digital revolution of the last century we now need a software revolution to aid spectroscopists in decoding the mountains of experimental data generated by biomedical vibrational spectra. This is where the full potential of artificial intelligence capabilities can be harnessed.

## 4. Data Mining the Raw Vibrational Spectra

The vibrational spectroscopic community has long been aware of the fact that the assessment of biomedical infrared and Raman spectra differs fundamentally from the conventional, long-established analysis of vibrational spectra generated from chemical or biological samples. Polyatomic molecules display a number of vibrational modes which are regarded as group frequencies and help in the analysis of vibrational spectra. For example, the infrared and Raman spectra of biomolecules like lipids have been successfully analyzed in terms of localized vibrations originating from the hydrophobic acyl chains and vibrations originating from the hydrophilic ester head groups. Similarly, the vibrational spectra of proteins have been interpreted in terms of their beta sheet or alpha helix domains as determined from the distinctive amide 2 bands that stem from the different hydrogen bonding pattern of the secondary protein structures.

The concept of “group frequency” which had been so useful for the interpretation of spectra obtained from chemical and biochemical specimens became of little use for the analysis of biomedical samples because spectra of cells and tissue are vastly more complex, containing information about many different biomolecules. Therefore, when it comes to the interpretation of infrared and Raman spectra obtained from biomedical specimens one must lean on more sophisticated data mining procedures. These computational methodologies include chemometrics, pattern recognition, artificial neural networks, genetic algorithms and many more. Biomedical vibrational spectroscopy has become highly dependent on these computational solutions which belong to the burgeoning fields of bioinformatics and medical informatics. Today practically all published studies that analyze biomedical vibrational spectra rely on biomedical informatics in some way or another. Astute spectroscopists have always known that there is more information in their vibrational spectra than could be obtained from the analysis of discrete bands. Now their insight can be satisfied.

The archetype of data mining is chemometrics, defined as the science of extracting information from chemical systems by data-driven means. Chemometrics ranges from overly simplistic to highly complex. An excellent overview of this field of multivariate statistical analyses can be found in the 2018 edition of “Chemometrics in Spectroscopy” by Mark and Workman [3]. Despite of having chemistry in its name, chemometrics is inherently interdisciplinary and like vibrational spectroscopy has expanded from chemistry to biology and also to medicine.

## 5. Where Are We Today? 

The historical evolution of biomedical vibrational spectroscopy is well documented [4,5]. From a technical point of view, applications of infrared and Raman spectroscopy in medicine can be sorted into three broad groups (i) the study of body fluids, (ii) the study of cells and tissues, and (iii) medical imaging, each requiring distinct models of analysis. From a medical perspective, vibrational spectroscopy can be applied to three main disciplines: (i) clinical spectroscopy (health care tests), (ii) spectral pathology (cyto- and histo-pathology) and (iii) spectral imaging (radiology). Biomedical infrared and biomedical Raman spectroscopic techniques have somewhat different strengths and complement each other the same way classical infrared and Raman spectroscopy do.

At this point the author would like to draw on his personal experience with biomedical vibrational spectroscopy. In 1992 the National Research Council of Canada established the Institute for Biodiagnostics in Winnipeg, an institute for molecular medicine dedicated solely to the non-invasive diagnosis of disease using NMR and vibrational spectroscopic techniques.

The group devoted to biomedical vibrational spectroscopy initially engaged in the fairly straightforward ex vivo analysis of such common body fluids as blood serum and urine [6]. The application of appropriate data mining routines to the analysis of the mid-IR spectrum of serum allowed the simultaneous quantitation of eight analytes: total protein, albumin, triglycerides, cholesterol, glucose, urea, creatinine and uric acid [7]. Similarly, the analysis of the near-IR spectrum of urine allowed the simultaneous quantitation of protein, creatinine and urea [8]. Building on these successes vibrational spectroscopic analysis was extended to other, less common body fluids such as synovial fluid for the diagnosis of arthritic conditions [9,10] and amniotic fluid for determining fetal lung maturity [11,12]. 

The early analysis of human blood serum and other body fluids by vibrational spectroscopy has paved the way for rapid expansion. Over the past 20+ years many studies were published investigating a variety of body fluids with a view to their eventual medical applications [13,14,15]. Meanwhile new research facilities dedicated to biomedical vibrational spectroscopy were established worldwide. Academic conferences and scientific organizations are now dedicated exclusively to this subject. A conference series titled “Shedding new light on disease” was started in Winnipeg in 2000 and continues to this day under the moniker SPEC. In 2015 the International Society for Clinical Spectroscopy, CLIR, was created to provide a platform dedicated to the translation of vibrational spectroscopy into the clinical environment. 

The vibrational spectroscopic analysis of human tissues, dubbed spectral pathology, turned out to be somewhat more difficult due to several confounding factors. For example, healthy tissue can be abnormal, yet not diseased. Additionally, morphology-dependent spectral distortions regularly encountered with human tissue samples continue to plague vibrational spectroscopists. The problems faced by spectral pathology have been documented [16,17] and new artefact removal algorithms are continually being developed [18]. Another obstacle to the wider application of vibrational spectroscopic pathology is the difficulty in obtaining sufficient reliable annotations. Correlating the IR and Raman spectroscopic features with tissue morphological features is a prerequisite for the training of trustworthy diagnostic algorithms.

The quest for the “holy grail” of spectral histopathology, the early detection of cancerous changes in tissues and cell smears, has motivated numerous scientists and research groups to turn to vibrational spectroscopy [19]. Their expectations stem from the understanding that the molecular motions studied by vibrational spectroscopy, often referred to as a “molecular dance”, are slightly different in diseased tissue compared to those in healthy tissue. By probing molecular vibrations of chemical bonds, vibrational spectroscopy reveals tissue biochemistry. Since disease leads to changes in tissue biochemistry before morphological or structural changes become visible, vibrational spectroscopy presents an effective tool for detecting and even staging the progression of disease. Naturally, the conversion of spectroscopic information into medically relevant information is heavily dependent on the use of appropriate algorithms supplied by biomedical informatics and AI.

Vibrational spectroscopic imaging is another fast-growing area of biomedical vibrational spectroscopy [20]. Notably, infrared imaging has received a major boost from the widespread use of military-grade focal plane array detectors, with numerous military uses now finding their way into medical applications. 

As an essentially non-invasive investigative methodology, vibrational spectroscopy is well suited to the study of living things, allowing in vivo spectral imaging of cells, tissues, and even parts of the body. An early application of in vivo imaging from our lab was the use of fuzzy C-means clustering and principal component analysis to study near-IR images taken of a human forearm during periods of venous outflow restriction and complete forearm ischemia [21]. 

Infrared and Raman spectroscopic imaging techniques are particularly fitting for applications in dermatology [22]. Future radiologists may develop AI surveillance algorithms for the early identification of suspicious skin lesions much like AI surveillance cameras are used today on public streets for facial recognition. The radiomic information obtained from vibrational spectroscopic images should be superior to that obtained from optical (visible) or x-ray images as it contains molecular type information. Vibrational spectroscopic imaging could become part of the multispectral imaging tool used by radiologists to extract “radiomic” information from images not discernible by visual inspection. This is not meant to replace radiologists, but to help them incorporate new tools into their practices. 

As in the case of body fluid analysis and spectral pathology, spectral imaging will only be as good as its underlying algorithms generated by biomedical informatics and AI. 

## 6. Biomedical Informatics Versus AI

At this point the question arises: what is the relationship between biomedical informatics and artificial intelligence? Traditionally the name bioinformatics was used for activities at the intersection of computer science and biological research, while the term medical informatics was used for research at the intersection of computer science and clinical medicine. More recently the term biomedical informatics has emerged to describe activities that bring together the disciplines of bioinformatics and medical informatics. The American Medical Informatics Association defines biomedical informatics as an “interdisciplinary field that studies and pursues the effective uses of biomedical data, information, and knowledge for scientific inquiry, problem solving, and decision making, motivated by efforts to improve human health” [23]. The European Commission has also produced a white paper focused on biomedical informatics [24], yet interestingly this document makes no mention of artificial intelligence. 

The problem with artificial intelligence is that it is a “catch all” label and different people understand different things under AI. The failed promises of the AI winter continue to haunt the field and many prefer to deliberately describe their work by other names such as informatics, knowledge-based systems or computational intelligence.

This author finds it peculiar that while machine learning, with its branches of supervised learning, unsupervised learning or deep learning is in fact part of AI, the term artificial intelligence is only rarely used in the vibrational spectroscopic literature. This is even more surprising since the term artificial intelligence is commonly used when describing innovative technologies in other fields of human activity, particularly in our societal interactions. The way AI systems use or rather abuse the limitless amount of personal information collected from search engines offered voluntarily by individuals through their Google, Facebook, Twitter, Instagram or other social media accounts, regularly makes headlines.

It is important to recognize that there is a difference between biomedical informatics and AI. Biomedical informatics is basically a tool, a tool used by the practitioners of biomedical spectroscopy for data mining. AI on the other hand is more than a tool, it is a collection of concepts, methods and programs which empower the different biomedical informatics packages. Figuratively speaking, biomedical informatics is a hammer, but this hammer is wielded by numerous AI paradigms and algorithms.

Biomedical informatics encompasses a number of key processes which transform data (meaningless raw symbols) to information (interpreted data with meaning), to knowledge (organized information), to intelligence (actionable knowledge). Biomedical informatics can also be impacted by AI indirectly. With the availability of specialized algorithms, it has become common for vibrational spectroscopists to apply off-the-shelf systems to mine and classify their experimental data. As more such commercial packages become available, researchers may face difficulties in choosing the most appropriate ones for their specific data set. AI based decision making has the potential to optimize the selection and yield the most appropriate software for each research study. With its ability to learn from past outcomes, AI could also predict the value of new software should the research environment change.

The move to digital medicine is largely driven by the industry’s adoption of emerging AI applications to healthcare. With its pattern recognition, decision making, learning and predictive algorithms, AI can probe large data sets that represent the electronic footprint of individual patients, leading to predictive analyses. Google Cloud Healthcare App for instance takes data from electronic health records through machine learning, creating insights for healthcare providers to make better decisions. Wearable healthcare technology like smart watches use AI to alert users and their health care professionals to potential issues and risks. Patient health assessment using AI technology eases the workload of professionals and prevents unnecessary hospital visits. 

As with any new technology entering the medical field, infrared and Raman based routines must integrate with current practices, gain regulatory approval and most importantly be embraced by the medical practitioners. Due to its interdisciplinary nature, practitioners of biomedical vibrational spectroscopy often struggle with the choice of the most appropriate platform to publish their work. Spectroscopic journals sometime find the topic “too medical” while medical journals find it “too spectroscopic” which has led to the emergence of new periodicals that cover this gap. Even though some of these journals have a more limited circulation, today’s AI-assisted search engines can find any pertinent publication.

## 7. Concluding Remarks

AI is rapidly transforming our society and revolutionizing many aspects of our lives. The power of AI is as broad a topic as it is a controversial one, spanning science and technology, socioeconomics, entertainment, politics and healthcare. The latter encompasses the present theme, namely the role of AI in exploiting vibrational spectroscopic data for the diagnosis and management of disease and other medical conditions. 

Today, a global community of teams is exploring the innovative use of vibrational spectroscopic techniques for point of care testing, advanced spectral histopathology and rapid in vivo diagnostics. AI-assisted biomedical vibrational spectroscopy is now poised to deliver an auxiliary platform for clinical analyses, histopathological examinations and radiological imagery. 

As we reflect on the success of past developments and on the rate of new innovations, we can predict that biomedical vibrational spectroscopy will embrace advances attained by artificial intelligence in other fields of human endeavor, eventually making it an indispensable instrument in the global medical toolbox of the 21st century. 

## Data Availability

Not applicable.
